# It is time to become serious about closing the global resource gap for FCTC implementation

**DOI:** 10.18332/tid/130809

**Published:** 2020-12-08

**Authors:** Ryan Forrest, Sara Rose Taylor

**Affiliations:** 1 Framework Convention Alliance for Tobacco Control, Ottawa, Canada

**Keywords:** FCTC, implementation, Official Development Assistance, COP9

Fifteen years on from the entry-into-force of the WHO Framework Convention on Tobacco Control (FCTC), it is time to reckon with the significant gap between FCTC policy guidance and action. One of the major barriers to progress on this front is the lack of sustainable and adequate resources.

Tobacco use kills over 8 million people each year, making it the leading preventable cause of death worldwide. While tobacco use prevalence has fallen in many high-income countries in recent decades, due in large part to the implementation of the FCTC, the FCTC Secretariat reports^1^ that 13% of Parties reported an increase in tobacco use prevalence between 2016 and 2018, while 24% reported no change.

Despite the fact that tobacco control measures are inexpensive and highly cost-effective, the political commitments embodied in the WHO FCTC and UN Sustainable Development Goals (SDG) Target 3.a have not yet translated into adequate financial commitments. In 2019, tobacco control programs received just US$66.23 million in development assistance for health (DAH); only 0.16% of all DAH^2^. Research Triangle Institute (RTI) International^3^ estimated that this level of international funding addresses 4% of estimated costs to scale up tobacco control to FCTC-compliant levels, indicating a US$27.4 billion resource gap. Domestic spending was also found to be insufficient to meet the level of need^3^.

Closing this gap requires action on multiple fronts. Domestic resource mobilization will have an important role to play. Increasing tobacco taxes presents a ‘win-win’ opportunity, raising revenue for health spending while reducing consumption. It will also be critical that governments prioritize domestic spending on tobacco control. FCA is currently supporting budget advocacy projects in Senegal and Uganda to build the capacity of national actors to make the case for investment in FCTC implementation to ministries in charge of budgeting for health.

International assistance will also be critical in supporting continued progress in low- and middle-income countries where domestic resources fall short. This includes an urgent need for more Official Development Assistance to support building sustainable capacity for tobacco control programs in these countries. There may also be scope to explore the role of innovative international financing arrangements, such as a multi-donor trust fund or vertical fund as seen under other treaty bodies.

Such investments in tobacco control measures may seem particularly challenging in the face of the resource constraints brought on by the COVID-19 pandemic, but they can support recovery and resilience by reducing health system burdens, creating healthier populations, and promoting sustainable development.

The FCTC Conference of the Parties (COP), as the world’s dedicated intergovernmental forum on tobacco control, is a key venue in which to discuss this problem and seek solutions. At next year’s 9th session of the FCTC COP, we would like to see a number of discussions taking place on how to mobilize additional resources to support treaty implementation. Key issues include:

- Regularly collecting and reporting data on domestic and international spending on tobacco control, including calculations of the global funding gap for FCTC implementation;
- Developing a clear investment case and fundraising strategy tied to the Global Strategy to Accelerate Tobacco Control;
- Providing additional assistance to Parties to support their domestic resource mobilization efforts.

## References

[cit0001] WHO Framework Convention on Tobacco Control (2018). 2018 Global progress report on the implementation of the WHO Framework Convention on Tobacco Control.

[cit0002] Institute for Health Metrics and Evaluation Financing Global Health 2019: Tracking Health Spending in a Time of Crisis.

[cit0003] Research Triangle Institute International Financing Gap to Implement Demand Reducing Tobacco Control Strategies in WHO FCTC Countries. https://www.fctc.org/wp-content/uploads/2019/02/FCTC-Funding-Gap-Report_9_18.pdf.

